# Risk factors for biochemical recurrence after robotic assisted radical prostatectomy: a single surgeon experience

**DOI:** 10.1186/s12894-015-0024-7

**Published:** 2015-04-08

**Authors:** Ryuta Tanimoto, Yomi Fashola, Kymora B Scotland, Anne E Calvaresi, Leonard G Gomella, Edouard J Trabulsi, Costas D Lallas

**Affiliations:** Department of Urology, Kimmel Cancer Center, Thomas Jefferson University, 1025 Walnut St. Suite 1112, Philadelphia, PA 19107 USA

**Keywords:** Biochemical recurrence, Biochemical recurrence free survival, Cox regression analysis, Positive surgical margin, Robotic assisted radical prostatectomy

## Abstract

**Background:**

Radical prostatectomy is a standard surgical treatment of clinically localized prostate cancer. Margin status has been found to be an independent predictor of biochemical recurrence (BCR) after open radical prostatectomy in several large series but this is still controversy in Robotic Assisted Radical Prostatectomy (RARP) series. We therefore wanted to investigate the prognostic significance of positive surgical margin (PSM) and other pathological factors on BCR in patients treated with RARP by a single surgeon.

**Methods:**

Prospectively collected data of 439 patients treated with RARP between October 2005 and June 2013 by a single surgeon at a single institution were analyzed. BCR was defined as follow-up PSA level > 0.2 ng/ml on two separate occasions or patients who had to undergo salvage therapy. Kaplan Meier curves and Log Rank test were used to compare the risk of BCR. Univariate and Multivariate Cox Regression analyses were performed to determine the prognostic impact of age, BMI, prostate weight, PSA prior to surgery, pathological T-stage, pathological Gleason sum, PSM and operative period.

**Results:**

In this study period, 34 out of 439 had BCR, giving an overall BCR rate of 7.7% for this cohort. Overall 2- and 3-year BCR-free survival rates were 93% and 88%, respectively. Patients with a PSM had a 2-year BCR free survival of 88% compared to 94% in those with negative margins (p < .0001). On the multivariate analysis, PSM as well as pathological Gleason sum > = 8, PSA, pathological stage and operative period were significantly associated with BCR.

**Conclusions:**

In our case series of RARP performed by a single surgeon, PSM as well as pathological Gleason sum, PSA, pathological stage and early operative period for this surgeon were the independent predictors of BCR.

**Electronic supplementary material:**

The online version of this article (doi:10.1186/s12894-015-0024-7) contains supplementary material, which is available to authorized users.

## Background

Radical retropubic prostatectomy (RRP) is a standard surgical treatment of clinically localized prostate cancer. Recently robotic-assisted radical prostatectomy (RARP) also has become very popular in the United States and Europe; it has been estimated that > 75% of radical prostatectomies are performed using the da Vinci platform (Intuitive Surgical, Inc., Sunnyvale, CA, USA) [1]. Systematic review of the literature revealed that RARP represented a safe procedure with better perioperative outcomes, such as reduced blood loss and postoperative hospital stay, when compared with open surgery [2,3]. Moreover, recent meta-analysis showed similar positive surgical margin (PSM) rates and biochemical recurrence (BCR) free survival estimates when comparing RARP with RRP and RARP with laparoscopic radical prostatectomy (LRP) [3]. At our institution, RARP was adopted in lieu of LRP in 2005 and our previous report also supported these findings [4].

Margin status is considered an independent predictor of BCR after open radical prostatectomy in several large series [5-7]. This was also seen in some robotic prostatectomy series [8-11]. However, in the largest reported robotic series with a median follow-up of 36 months, margin status was not shown to be an independent BCR predictor [12].

The aim of this study was to assess the prognostic significance of PSM and other pathological factors on BCR in patients treated with RARP by a single surgeon.

## Methods

A single institution retrospective review of RALP performed by a single surgeon between October 2005 and June 2013 was performed. This is a Thomas Jefferson University Institutional Review Board approved database (approval reference: 02.9000) in which data has been collected prospectively. The written informed consent for participation in the study was obtained from all patients. Patients were initially evaluated at a multidisciplinary clinic. Of 1062 consecutive patients who underwent RARP in our institution, a total of 561 patients were treated by a single surgeon (EJT) during this time. Following the exclusion of patients who did not have recorded PSA values postoperatively (n = 73) or had adjuvant radiation or hormonal treatment (n = 9), who had positive lymph node (n = 2), pT3b (n = 2), pT3a with positive surgical margin (n = 3), pT3a with tertiary GS 5 (n = 1) or high GS (4 + 5) with positive surgical margin (n = 1), the remaining 439 patients were evaluated in the present study. None of these patients had been administered hormones prior to surgery. All prostate specimens were submitted in their entirety and underwent standard whole mount step sectioned pathologic analysis in order to determine surgical Gleason score, pathological stage and margin status. The location of each positive margin on the prostatic specimen was examined. A confirmatory second level pathologic review with a genitourinary pathologist and the surgical team was performed weekly in a multidisciplinary genitourinary pathology conference. BCR was defined as follow-up PSA level > 0.2 ng/ml on two separate occasions or patients who had to undergo salvage therapy. Kaplan Meier curves and Log Rank Test were used to compare the risk of developing BCR. Univariate and Multivariate Cox Regression analyses were performed to determine the prognostic impact of pathological factors including age, BMI at surgery, pre-operative PSA (< 10 ng/ml versus > = 10 ng/ml), operative period (early operative period for this surgeon, 2005 – 2007 and later operative period 2008 – 2013), PSM, foci of PSM (unifocal versus multifocal versus none), pathological stage (T2 versus T3/4), pathological Gleason sum (<= 6 versus 7 versus > = 8), extracapsular extension (unifocal versus multifocal versus none), seminal vesicle involvement, perineural invasion, and prostate size as determined by weight in grams.

All procedures were performed by a single surgeon (EJT) using the da Vinci® Surgical System. Laparoscopic ports were placed using a 6-port transperitoneal approach. The seminal vesicles were approached posteriorly. Nerve sparing procedures were attempted for all patients with appropriate preoperative potency and acceptable oncologic risk. For the initial 50 RARP patients, obturator lymphadenectomy was performed if the preoperative Kattan nomogram [13] predicted greater than 1% risk of lymph node invasion. Subsequently, all patients were treated with obturator node dissection with high risk patients, as determined by the D’Amico criteria [14], receiving extended lymphadenectomy to include external iliac nodes.

### Statistical analysis

All statistical analyses were two-tailed. Differences were considered significant if the p value was < 0.05. The statistical analysis was conducted with JMP version 9.0 (SAS Institute Inc, Cary, NC, USA).

## Results

Out of 531 patients treated by a single surgeon, 439 were included in this study. The clinical and pathological characteristics of the 439 patients are listed in Table 1. Median patient age at prostatectomy was 59 years. Median PSA was 4.9 ng/ml (interquartile range, (IQR) 3.9 – 6.3). Overall, 422 patients (96%) underwent lymph node dissection with a median rate of 7 lymph nodes (IQR 4 – 12). Among those, 4 patients (0.9%) had at least 1 positive node. The median follow-up time was 16 months (IQR 6 – 34). In this study, 34 of the 439 follow-up patients (7.7%) experienced BCR. Among those, 31 (91%) BCR were due to elevated PSA recurrence and only 3 received salvage radiation therapy before a documented PSA increase. All 20 patients with secondary treatment had salvage radiation therapy with or without hormonal therapy. In all, 119 patients (27.1%) had PSM and among those, 102 (85.7%) were unifocal. The locations of PSM (Additional file 1: Table S1) were posterolateral (54.6%), bladder neck / base (14.3%) and apex (10.9%). The PSM rates were 20%, 49% and 50% in patients with stage pT2, pT3a and pT3b respectively. Unfortunately, we failed to identify improvements in the PSM over time (Additional file 2: Table S2).

**Table 1:** **Patient characteristics (n = 439)**

				**BCR(+)**	**n = 34**	**BCR(−)**	**n = 405**
	**Median**		**IQR**	**Median**	**IQR**	**Median**	**IQR**
Age	(years)	59	55-65	62	56-66	59	55-64
BMI	(kg/m^2^)	28.1	25.6-31.2	29.3	25.5-33.2	28	25.7-31.1
Preoperative PSA (ng/ml)		4.9	3.9-6.3	6.7	4.5-13.2	4.9	3.9-6.0
Follow up time (months)		16	6-34	33	3-49	15	6-33
		n	%	BCR(+)	n = 34	BCR(−)	n = 405
Clinical Stage							
	T1c	342	77.9%	22	64.7%	320	79.0%
	T2a	65	14.8%	5	14.7%	60	14.8%
	T2b	24	5.5%	5	14.7%	19	4.7%
	T2c	7	1.6%	2	5.9%	5	1.2%
	T3a	1	0.2%	0	0.0%	1	0.2%
Clinical Gleason							
	≤6	216	49.2%	8	23.5%	208	51.4%
	7	202	46.0%	21	61.8%	281	69.4%
	≥8	21	4.8%	5	14.7%	16	4.0%
Pathological Stage							
	T2a	50	11.4%	0	0.0%	50	12.3%
	T2b	7	1.6%	0	0.0%	7	1.7%
	T2c	280	63.8%	10	29.4%	270	66.7%
	T3a	75	17.1%	12	35.3%	63	15.6%
	T3b	24	5.5%	9	26.5%	15	3.7%
	T4	3	0.7%	3	8.8%	0	0.0%
Pathological Gleason							
	≤6	157	35.8%	0	0.0%	157	38.8%
	7	241	54.9%	6	17.6%	235	58.0%
	≥8	41	9.3%	28	82.4%	13	3.2%
Operative period							
	2005-2007	102	23.2%	14	41.2%	88	21.7%
	2008-2010	203	46.2%	13	38.2%	190	46.9%
	2011-2013	134	30.5%	4	11.8%	130	32.1%
Positive Surgical Margin		119	27.1%	20	58.8%		
BCR		34	7.7%	34	100.0%		
	PSA > 0.2	31	7.1%	31	91.2%		
	SalvageXRT	20	4.6%	20	58.8%		

**Figure 1:**
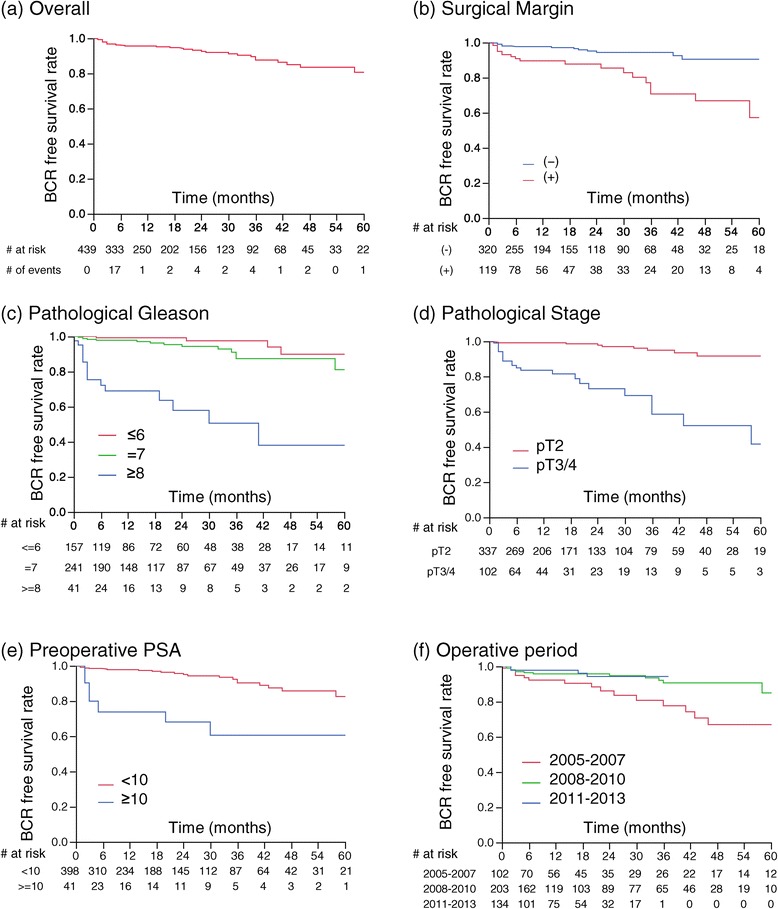
**Kapalan-Meier curves. (a)** overall, **(b)** Biochemical recurrence free survival (BCRFS) in patients with (red curve) and without positive surgical margins (blue curve). **(c)** BCRFS for pathological Gleason < =6(red curve), =7(green curve) and > =8(blue curve). **(d)** BCRFS for pathological stage pT2 (red curve), pT3-4 (blue curve). **(e)** BCRFS for preoperative PSA < 10 (red curve), preoperative PSA > =10. **(f)** BCRFS in patients operated in 2005–2007 (red curve), in 2008 - 2010(green curve), in 2011–2013 (blue curve).

Overall 2-, and 3-year BCR-free survival (BCRFS) rates were 93% and 88%, respectively (Figure 1a). Patients with a PSM had a 2-year BCRFS of 88% compared to 94% in those with negative margins (Figure 1b; p < 0.0001). The two year BCR free rate was 99%, 94% and 58% for patients with pathological Gleason sum < = 6, 7 and > = 8, respectively (Figure 1c; p < 0.0001); the same rate was 98% and 73% in patients with pT2 disease and with pT3/4 respectively (Figure 1d; p < 0.0001). Preoperative PSA > = 10 (Figure 1e; p < 0.0001), and early operative period, 2005 – 2007 (Figure 1f; p = 0.0093), which was the period during which the first RARPs were performed by this surgeon, were also significantly associated with increased risk of BCR.

Table 2 summarizes data of the univariate and multivariate analyses for predictors of BCR. On univariate analysis, BMI, pathological Gleason sum > = 8, pathological stage, PSM, the foci of PSM, PSA and operative period were significantly related with BCR. Extracapsular extension (unifocal versus multifocal versus none), seminal vesicle involvement and perineural invasion were also related with BCR (p < 0.0001, p < 0.0001 and p = 0.0004, respectively; data not shown). On multivariable analysis, pathologic Gleason sum was the strongest predictor of BCR, with an HR of 6.76 (p = 0.0030) for Gleason > = 8 when compared to Gleason < = 6. The presence of PSM also represented independent predictors of BCR (HR 2.69; p = 0.0153) as well as PSA, pathological stage (HR 4.48; p = 0.0011) and early operative period (HR 0.38; p = 0.0113).

**Table 2:** **Univariate and multivariate analyses of factors affecting BCR**

	**Univariate analysis**			**Multivariate analysis**		
	**Risk ratio**	**(95% CI)**	**p-value**	**Risk ratio**	**(95% CI)**	**p-value**
Age	1.03	(0.98-1.09)	0.2279	0.98	(0.92-1.05)	0.6476
BMI	1.09	(1.02-1.17)	0.0158	0.97	(0.90-1.04)	0.3369
Prostate Weight (gm)	0.99	(0.96-1.01)	0.2851	-		
PSM	4.12	(2.09-8.34)	<0.0001	2.69	(1.21-6.14)	0.0153
PSM foci						
None	1.00			-		
Unifocal	3.49	(1.67-7.32)	0.0010	-		
Multifocal	9.23	(2.96-24.34)	0.0005	-		
Pathological Gleason						
≤6	1.00			1.00		
7	2.39	(0.86-8.42)	0.1001	1.78	(0.58-6.63)	0.3226
≥8	21.11	(7.71-73.90)	<0.0001	6.76	(1.87-29.21)	0.0030
Preoperative PSA						
<10	1.00			1.00		
≥10	6.66	(3.18-13.28)	<0.0001	2.53	(1.06-5.81)	0.0360
Pathological Stage						
pT2	1.00			1.00		
pT3-4	10.89	(5.33-23.98)	<0.0001	4.48	(1.83-11.30)	0.0011
Operative period						
Before 2007	1.00			1.00		
After 2008	0.36	(0.18-0.72)	0.0045	0.38	(0.18-0.80)	0.0113

BMI; body mass index, PSM; positive surgical margin.

## Discussion

In our case series of RARP performed by a single surgeon, positive surgical margin as well as pathological Gleason sum, PSA, pathological stage and early operative period were the independent predictors of BCR.

Most RARP studies report short-term (< 12 months) follow-up outcomes, though 4 large studies recently reported BCR free survival (BCRFS) after RARP with a follow-up of more than 5 years [10,15-17]. The largest report of PSA outcomes in the RARP literature is from Menon et al. [16], who reported an overall BRFS of 86.4% for 1384 patients with a median follow-up of 60.2 months. Actual 3-and 5-yr BCRFS were 90.6% and 86.6%, respectively. On the other hand, Suardi et al. and Liss et al. reported 3- and 5-year BCRFS of 94%, 86% in 184 patients and 87.8%, 84.9% in 435 patients, respectively. In our study, mean follow-up was only 22 months and it is too early to define 5-year BCRFS, but the overall 2-year and 3-year BCRFS was 93% and 88%, respectively, which was comparable to previous reports.

The overall PSM rate in this series is 27.1% with a rate of 19.9% for pT2 tumors. This is comparable to other contemporaneous RARP series whose PSM rates were 6.5 - 29.5% overall and 2.5 - 22.7% in patients with stage pT2 [8-11,16-26]. Regarding risk factors for BCR, all four large studies referenced above agreed that the pathological Gleason score was an independent factor, but two of them did not find that the presence of positive margins was significant on multivariate analysis. Menon et al. showed the significance of positive margin on BCR, although their previous series with follow-up of 36 months did not [12]. The reason for this may be that the actual BCR rate (2.4%) was too low to power the statistical significance in this cohort. Sooriakumaran et al. reported an RARP case series of 944 patients with median follow-up of 6.3 years, which showed that PSM status as well as preoperative PSA > 10, pathological Gleason sum > = 4 + 3, pathological T3 disease and lower surgeon case volume were all associated with increased risk of BCR on multivariable analysis.

Shikanov et al. reported not only the presence of PSM but also PSM length (> 3 mm) to be independently associated with BCR. Interestingly, patients with negative margins and those with a positive margin less than 1 mm had similar rates of biochemical recurrence [9]. This finding is in keeping with others and suggests PSM > 3 mm and multifocal positivity were associated with risk of BCR [27]. Moskovic et al. reported that high body mass index does not affect BCR following robotic assisted laparoscopic prostatectomy when BMI was stratified into 3 groups (> = 30, > = 25 and < 30, < 25), although there was a trend toward increased recurrence in the obese [28]. In the present study, higher BMI had higher BCR on univariate analysis, but not on multivariate analysis.

With regard to the effect of surgeon experience on BCR, Zorn et al. [29] demonstrated that the risk of PSA recurrence was quite stable over 700 cases when compared with 3 groups (cases 1–300, 301–500, and 501–700). Samadi et al. also assessed the effect of surgeon experience and technical modifications, which were categorized as initial, intermediate and current technique, on oncological outcome after RARP. Pathological T2 margin rates decreased continuously during the initial technique period, followed by a transient worsening of margin rates during the intermediate time period and a subsequent decrease during the period when the current technique was used, but no significant differences were noted in BCR rate between these groups. In both studies, follow-up duration was relatively short, and BCR-free survival analyses adjusted for covariates were not provided. In the present study, we adjusted BCR-free survival for the covariates including pathological factors with Cox Regression analysis. The early operative period, 2005–2007, which encompassed the first 100 cases, had a significantly higher rate of BCR compared to the late period, but there was no difference between 2008 – 2010 and 2011 – 2013. This suggested the risk of BCR was stable after 100 cases.

It is also important to remember that BCR does not necessarily lead to clinical recurrence or cancer specific mortality, and BCR without clinical progression might reflect the recurrence of indolent prostate cancer or the presence of benign prostatic tissue left behind after surgery [30]. Hence, it is necessary to follow up our cohort further and determine the impact of BCR on longer-term oncologic outcome.

Our study has some limitations. Many of the patients were from outside our geographic area and are followed locally. The median follow-up in these patients was 16 months, and so these results must be considered early. In addition, factors potentially correlating with BCR, such as length of PSM were not included in this analysis. The main strength of our study is that only patients treated by a single surgeon were selected for this analysis which was adjusted for the operative periods with the aim of decreasing the influence of surgeons’ techniques on their outcomes.

## Conclusions

In our case series of RARP performed by a single surgeon, positive surgical margins as well as pathologic Gleason sum, PSA, pathologic stage and early operative period were the independent predictors of BCR. Further follow-up is necessary to determine how this finding will translate into cancer-specific and overall survival outcome.

## Additional files

Additional file 1: Table S1. The margin location in the patients with positive surgical margins (n=119). Additional file 2: Table S2. The positive surgical margin rate for each pathological stage over time.

## Abbreviations

BCR: Biochemical recurrence
RARP: Robotic Assisted Radical Prostatectomy
PSM: Positive surgical margin
PSA: Prostate specific antigen
BCRFS: Biochemical recurrence free survival
IQR: Interquartile range
